# Anti-dipeptidyl-peptidase-like protein-6 (DPPX) autoimmune encephalitis associated with severe multifocal dystonia

**DOI:** 10.1016/j.prdoa.2025.100320

**Published:** 2025-04-02

**Authors:** Eric Roddy, Erik Gentry, Victoria Holiday, David Robertson, Peter Hedera

**Affiliations:** a University of Louisville School of Medicine, Department of Neurology, USA; b Norton Neuroscience Institute, Department of Neurology, University of Louisville, 220, Abraham Flexner Way, Suite 606, USA

**Keywords:** Dystonia, Autoimmune encephalitis, DPPX, Immunotherapy, Toxin therapy

## Abstract

Anti-DPPX encephalitis is a rare form of autoimmune encephalitis characterized antibodies against a subunit of Kv4.2 potassium channels. Characteristic clinical features include cognitive dysfunction, parasomnias, psychosis, and seizures. Motor symptoms typically include myoclonus, tremor, and midline ataxia. DPPX encephalitis presenting as new-onset focal dystonia has not been previously described.

## Highlights

• Anti-DPPX encephalitis is characterized by complex symptoms, including movement disorder.
• Dystonia has not been described as a phenomenon of anti-DPPX encephalitis.
• Immunosuppressive therapy is the mainstay of treatment for anti-DPPX encephalitis.
• Botulinum toxin administration may improve the symptoms of dystonia in anti-DPPX encephalitis.

## 1 Introduction

Autoimmune encephalitis is a heterogenous group of disorders most commonly characterized by neurocognitive decline, seizures, psychiatric symptoms with hallucinations and delusions, and sleep disturbances [1,2]. Movement disorders are other possible manifestations of autoimmune encephalitis, and chorea, dyskinesias, ataxia, myoclonus, and tremor are typically described in these conditions [[1], [2], [3], [4]]. Distinct motor symptoms may be suggestive for a specific type of causative antibodies, such as orofacial-lingual dyskinesia with N-methyl-D-aspartate receptor encephalitis [2]. Dystonia is relatively rarely observed in patients with autoimmune encephalitis with a notable exception of LGI1 encephalitis, where faciobrachial dystonic seizures are essentially diagnostic for this entity.

Anti-dipeptidyl-peptidase-like protein-6 (DPPX) encephalitis is a rare form of autoimmune encephalitis characterized by the presence of antibodies against DPPX, a subunit of Kv4.2 potassium channels [5]. Characteristic clinical features include rapid onset of cognitive problems, parasomnias, psychosis with hallucinations and seizures [5]. Anti-DPPX autoimmune encephalitis typically presents with a distinctive pattern of symptom onset, characterized initially by prominent gastrointestinal manifestations including severe diarrhea and weight loss. Over time, patients develop progressive neurological symptoms, which typically begin with subtle cognitive changes and sleep disturbances before advancing to more severe manifestations such as memory impairment, movement disorders, and speech difficulties [4]. Motor abnormalities typically include myoclonus, mild postural tremor and mild midline gait ataxia. With disease progression, patients may experience myoclonus, hyperekplexia, autonomic dysfunction, and seizures. This temporal evolution from predominant gastrointestinal symptoms to multisystem neurological involvement is a hallmark feature that can aid in early recognition of this rare but treatable autoimmune condition [5].

Dystonia has not been reported as a feature in DPPX encephalitis. Here we report a patient diagnosed with DPPX encephalitis who also developed severe multisegmental dystonia, further widening the clinical phenotype of this autoimmune encephalitis.

## 2 Case Report

We describe a 35-year-old male who presented with a three-year history of visual hallucinations, cognitive decline, gait disturbance, and parasomnias with severe insomnia. These symptoms were preceded by chronic diarrhea and an approximate 15 kg weight loss. His neurologic examination showed midline gait ataxia and a mild bilateral postural arm tremor. Family also reported occasional muscle jerks in early stages of his disease, but they were never observed during multiple neurologic examinations. The patient’s level of functionality deteriorated to a level requiring near-total care by his family. Approximately two years after the onset of his symptoms he also developed cervical dystonia with predominant head turning to left and tilting to the right. Retrocollis was also present (Video S1 and S2). He also exhibited bilateral hand dystonia with wrist flexion. He had a baseline Toronto Western Spasmodic Torticollis Rating Scale (TWISTER) severity score of 24; he had no exposure to dopamine-blocking agents. Myoclonic jerks, startle reaction, or tremor was not observed during repeated neurologic examinations.

Initial autoimmune and reversible encephalopathy workup was negative. MRI was unremarkable, and metabolic PET scan showed patchy areas of reduced uptake in both frontal lobes. Over the course of 64 h, EEG monitoring did not identify epileptic activity. However, there was absence of sleep activity over the entirety of the recording period. CSF studies demonstrated seven nucleated cells, normal total protein and the presence of four oligoclonal bands. An initial paraneoplastic/autoimmune encephalitis panel did not reveal evidence of disease. Extended reanalysis of the paraneoplastic panel, all performed at the Mayo Clinic Laboratories, revealed the presence of anti-DPPX antibodies, and cell-based assay showed a high positive anti-DPPX antibody titer. Creatine kinase (CK) levels were never elevated.

Treatment with cyclophosphamide, and later with rituximab, resulted in improvement of the patient’s cognitive and psychiatric symptoms. He completed six infusions of cyclophosphamide at the dose of 1140 mg (600 mg/m^2^), followed by Rituximab 1000 mg every six months, with three completed cycles so far. However, this has not improved his dystonia, thus therapy with botulinum A toxin was initiated. A treatment regimen of 1000U total of botulinum A toxin with 500U used for cervical dystonia and 500U utilized for bilateral hand dystonia significantly improved his cervical and limb dystonia. Ongoing treatment was continued with botulinum toxin every three months and with rituximab every six months. Immunosuppression resulted in lack of symptom progression.

Family history was negative for any neurologic problems, including dystonia in all first- and second-degree relatives. Both parents of the proband were examined by the corresponding author, and their examination was unremarkable.

## 3 Discussion

Anti-DPPX encephalitis has distinct clinical features of initial gastrointestinal problems and weight loss, followed by neurocognitive and psychiatric symptoms [5]. However, this entity currently lacks defined diagnostic criteria and treatment guidelines and requires a high index of suspicion. Movement abnormalities are relatively common but quite non-specific. DPPX encephalitis presenting as new-onset focal dystonia has not been previously described. Our report expands upon motor phenomenology that may be associated with this autoimmune condition.

Myoclonus and hyperekplexia are most typical core motor features of DPPX encephalitis [6]. CNS hyperexcitability is a prominent pathophysiologic phenomenon in most patients, and this may be associated with additional muscle rigidity [7]. Our patients did not demonstrate myoclonus or startle reaction, but he exhibited a typical cervical and limb dystonia with involuntary, sustained, or repetitive muscle contractions causing abnormal postures. Interestingly, his family reported some jerking movement early in the course of the disease, but they were never observed during our examinations. The reason why our patient developed more dystonic phenomenology rather than hyperkinetic myoclonus is unknown. However, pathogenesis of dystonia also involves cortical hyperexcitability, and it is possible that additional basal ganglia involvement may be the reason for more pleomorphic motor phenomenology in our patients.

Management of DPPX encephalitis primarily focuses on treating the underlying autoimmune condition with immunotherapy [5]. Our patient experienced improvement of symptoms except for multifocal dystonia. Standard therapeutic approach with botulinum A toxin therapy resulted in control of dystonic symptoms.

The presence of dystonia along with other neurological symptoms that are preceded by gastrointestinal problems may help differentiate DPPX encephalitis from other conditions in the future as more cases are recognized. However, the prevalence of dystonia in DPPX encephalitis remains unknown and this report should increase the awareness of this symptom in this autoimmune encephalitis.

## 4 Ethical Compliance Statement

All procedures performed in studies involving human participants were in accordance with the ethical standards of respect for persons, beneficence, and justice. As this was a case study, IRB oversight was not necessary. Written and verbal consent was granted by the patient and his medical decision maker, his mother, for the use of his deidentified data, pictures, and videos. Measures were taken to eliminate identifiable patient features such as his head, face, and eyes. We confirm that we have read the Journal’s position on issues involved in ethical publication and affirm that this work is consistent with those guidelines.

## CRediT authorship contribution statement

**Eric Roddy:** Writing – review & editing, Writing – original draft, Visualization, Project administration, Investigation, Conceptualization. **Erik Gentry:** Writing – review & editing, Visualization, Conceptualization. **Victoria Holiday:** Writing – review & editing, Supervision. **David Robertson:** Writing – review & editing, Investigation. **Peter Hedera:** Writing – review & editing, Writing – original draft, Supervision, Project administration, Investigation, Conceptualization.

## Declaration of competing interest

The authors declare that they have no known competing financial interests or personal relationships that could have appeared to influence the work reported in this paper.
